# Shared decision-making for patients with psychotic disorders: validating the measure “collaboRATE for Next of Kin acting to support patient autonomy”

**DOI:** 10.3389/frhs.2026.1887589

**Published:** 2026-09-30

**Authors:** Roar Fosse, Irene Norheim, Jan Ivar Røssberg, Maria Romøren, Kristin Sverdvik Heiervang, Lars Hestmark, Reidar Pedersen

**Affiliations:** 1 Division of Mental Health and Addiction, Vestre Viken Hospital Trust, Drammen, Norway; 2 Institute of Clinical Medicine, University of Oslo, Oslo, Norway; 3 Centre for Medical Ethics, University of Oslo, Oslo, Norway; 4 Division of Mental Health Services, Akershus University Hospital, Lørenskog, Norway

**Keywords:** caregivers, collaboRATE measure, family involvement, shared decision-making, supported decision-making

## Abstract

**Introduction:**

Family involvement and shared decision-making (SDM) are key elements in current mental health treatment. While it is important to measure the level of SDM from the perspective of relatives, suitable validated measures are lacking. Moreover, supported decision-making has gradually been emphasized more than substituted decision-making. In a large-scale implementation study of family involvement in the treatment of psychosis, we explored SDM for patients’ closest relatives. We present the first validation of a three-item instrument to evaluate SDM for relatives acting to support patient autonomy, building on existing collaboRATE measures

**Methods:**

For the new collaboRATE measure for relatives, we assessed scale reliability and concurrent and discriminant validity using available data from our implementation study. The study included relatives of patients treated for psychosis at community mental health centers, with data acquired and analyzed at baseline (*n* = 198 relatives) and 6-month follow-up (*n* = 173)

**Results:**

The new collaboRATE measure for relatives had excellent scale reliability, with Cronbach's alpha values ranging from 0.91 to 0.95. Scores on the collaboRATE for relatives were correlated with the scores on matching items from the Carer Well-Being and Support questionnaire, with Pearson *r* values varying from *r* = 0.49 to *r* = 0.66, supporting concurrent validity. Exploratory analysis provided further tentative support for concurrent validity, indicating significantly lower collaboRATE scores for relatives of patients in compulsory care compared to voluntary care. We observed small negative correlations between the collaboRATE scores and the relatives’ responses to subscales of the Experience of Caregiving Inventory, which only partly supported discriminant validity

**Conclusion:**

These validation data provide initial support for the new collaboRATE measure to assess SDM from the perspective of relatives supporting patient autonomy, although further validations are needed.

## Introduction

The involvement of relatives in the treatment of patients with severe mental illness provides a range of benefits for both parties. For patients with psychotic disorders, this includes reduced relapse rates and hospitalizations, as well as improved functioning. Benefits for relatives include reduced psychological distress, caregiver burden, and expressed emotions, along with improved quality of life (1–3). Family psychoeducation (FPE) is the most well-documented family intervention for individuals with psychotic disorders (4–6).

In a large-scale controlled study that monitored the effects of implementing family involvement in the treatment of patients with psychotic disorders, the IFIP study (7), we focused on patients’ and relatives’ appraisal of shared decision-making (SDM). In the IFIP study, a focus on SDM was a natural extension of relatives’ involvement, with the two approaches sharing several factors, including a long-term perspective, collaboration and recovery-oriented thinking, while emphasizing patient autonomy (8).

SDM refers to a model of health communication that encourages an egalitarian approach to decision-making, recognizing the skills and experience offered by all involved participants (9, 10). Central to SDM is the collaborative work between at least the patient and a provider, the sharing of information and exploration of health concerns, the discussion of treatment options and preferences, and agreed-upon decisions about courses of action and implementation (11). While policymakers and researchers in mental healthcare services initially focused on the importance of SDM for patients (12), several authors have suggested that caregiver involvement could be an integral part of and enhance SDM (13, 14). Caregivers often wish to be accepted as partners by mental healthcare providers and be given the opportunity to share their expertise (8).

Traditionally, relatives have been given legal rights primarily to be involved in healthcare as substitutes or surrogate decision makers if an adult patient lacks competence to consent, and in cases of coercive mental healthcare (15). Moreover, legislation often gives parents the right to consent to healthcare on behalf of their children if the child is below the legal age to consent. Propelled by the Convention on the Rights of Persons with Disabilities (CRPD) (16), which entered into force in 2008, there has been a gradual shift from substituted or surrogate decision-making to supported decision-making, both in healthcare and in welfare law more generally, and a stronger focus on removing special and potentially discriminatory regulations on coercion for patients with mental illness. Importantly, in some Western countries including Norway, surrogate decision-making has never been implemented in health law (17, 18). This means that when adult patients lack the competence to consent, relatives are not entitled to act or consent on their behalf. Instead, the relatives’ role has been to support the patient's autonomy, for example, by informing the clinician of what the patient would have wanted if they were competent (18). Furthermore, the need for decision-making support and family involvement for patients with severe mental illness extends beyond situations where the patient may lack the competence to consent. Moreover, the implementation of evidence-based family interventions is often lacking (19). One often-mentioned barrier to family involvement is that clinicians fear that involvement of relatives may undermine their alliance with the patient (20). One way to assess this alliance is to measure the level of SDM from the patient's perspective.

In the IFIP study, we used a triadic approach to family involvement, where the triad consisted of the patient, the relative(s), and the clinician(s), independent of whether the patient was competent to consent to treatment. To better address clinicians’ concerns about the possible loss of alliance, we assessed whether triadic interventions could improve SDM for relatives without reducing it for patients. However, doing so required us to measure the level of SDM from the perspective of relatives, both in situations where the patient could make autonomous decisions (eventually with some support from the clinician and the closest relative) and in periods where this ability was lacking. Most patients with longstanding, severe mental illness are competent to consent, but this may fluctuate and depend on the quality of supported decision-making.

Measures have been developed to assess the level of SDM from the patient perspective and from the perspective of parents and relatives acting as surrogate decision makers, where some of the patient measures have been validated (21–23). Such short, comprehensible, and valid measures are important for assessing healthcare quality and provider performance in both routine healthcare delivery and research. To our knowledge, no validated measure assesses SDM from the perspective of relatives acting to support patient autonomy (22, 24, 25), regardless of whether the patient is competent to consent to treatment. This is a paradox given the increasing political emphasis on shared decision-making and the need for family carers to support patients with severe and long-standing illness. To study SDM from the perspective of relatives supporting patient autonomy, we developed the measure “collaboRATE for Next of Kin Acting to Support Patient Autonomy.”

This study aimed to examine the reliability and validity of the new measure of SDM for relatives. Our research questions were as follows:

1. What is the reliability (or internal consistency) of the new collaboRATE scale for relatives?
2. What is the concurrent validity of the new collaboRATE scale for relatives in terms of correlations with relatives’ scores on items of the Carer Well-Being and Support questionnaire (CWS) part B, which measures components similar to the SDM construct?
3. To what extent does the new collaboRATE scale for relatives demonstrate discriminant validity, as revealed by weak and non-significant correlations with subscale scores on the Experience of Caregiving Inventory (ECI), which we hypothesized to be unrelated constructs?
4. Explorative on concurrent validity: Do relatives of patients in compulsory care score lower on collaboRATE than relatives of patients in voluntary care?

## Materials and methods

### Design

We used relevant data from the IFIP study, a cluster randomized trial in which family involvement approaches were implemented at 15 community mental health center (CMHC) units in Norway (7). The IFIP study focused on improving clinicians’ involvement and support of relatives, thereby contributing to patients’ and their relatives’ psychosocial health, quality of life, and service satisfaction. The study included patients with psychotic disorders and a close relative—designated by the patient as a spouse, parent, or any other close person—who has legal rights regarding the health care services.

In this validation study, we analyzed data reported by relatives across the two study arms at two time points: baseline and 6-month follow-up. We conducted all reliability and validity analyses at both time points, providing two parallel and overlapping yet distinct investigations of the research questions. Although the trial also collected data at the 12-month follow-up, fewer than half of the relatives had relevant data at this time point, so we excluded these data from this sub-study.

### Participants

The IFIP trial included patient/ relative pairs where the patient had a diagnosis of psychosis. Not included in the trial were patient-relative pairs where the patient was judged not competent to consent. Active psychosis was not automatically considered to make patients incompetent to participate. Rather, competence to consent to participate in the IFIP trial had to be assessed individually by the clinician recruiting the patient. The trial did not collect information regarding whether participating relatives had a formal legal standing as “closest relative” or had been designated to support the patient in clinical decision-making in the event of loss of decision-making capacity (7). Relatives with extensive previous participation in family psychoeducation were excluded. Using these criteria, the IFIP trial included 231 patient/relative pairs in treatment for psychosis at the CMHC units.

In this sub-study, we excluded relatives who reported no contact with their CMHC during either the 12 months prior to baseline or the period between baseline and 6-month follow-up. This led to the inclusion of 213 of the 231 relatives (92%) in the analysis. Of the 213 included relatives, 198 (93%) had data at baseline and 173 (81%) had data at 6-month follow-up. The baseline and 6-month subsamples had 159 relatives (75%) in common, 39 relatives were included only in the baseline group, and 15 were included only in the 6-month group.

The participant group of relatives (*n* = 213) consisted of 66.5% women and 33.5% men, with a mean age of 54.4 years. Regarding their relationship with the patients, 65.4% were parents, 14.3% were married to or cohabited with the patient, 10.8% were siblings, 6.5% were children, and five were relatives with other relationships to the patient. Patients’ clinicians reported that at baseline, 19 patients were in compulsory mental healthcare without an inpatient stay; we included relatives of all these patients in this sub-study.

### Variables and measures

#### The new collaboRATE measure for shared decision-making for relatives

To measure SDM, Elwyn et al. first developed the collaboRATE version for patients. The patient version consists of three questions: (1) How much effort was made to help you understand your health issues? (2) How much effort was made to listen to the things that matter most to you about your health issues? (3) How much effort was made to include what matters most to you in choosing what to do next? The questions are answered on a 10-point Likert scale ranging from 0 (no effort was made) to 9 (every effort was made), referring to respondents’ contact with mental healthcare personnel at the CMHC unit during the previous 6 months (23, 26). The original patient version of collaboRATE also lends itself to routine clinical use and has excellent discriminant and concurrent validity, inter-rater reliability, and sensitivity to change (27).

When developing the new collaboRATE version for relatives who act to support patient autonomy, we started with a Norwegian translation of the patient version (28) and a US collaboRATE version for individuals acting on behalf of patients, i.e., as a substitute or surrogate decision maker (29). We translated the US surrogate decision-maker version into Norwegian, adhering to the collaboRATE translation protocol (30). Because the original US version was created for surrogate decision makers and emphasized what mattered most to the surrogate, we slightly modified two items to emphasize what the relative regarded as most important for the patient. These changes were made to comply with legislation and scenarios where relatives are not entitled to act or consent on behalf of the patient, and to adopt an approach emphasizing supported decision-making rather than surrogate decision-making, without disregarding what relatives think is important. The translation and adaptations were thoroughly discussed within our interdisciplinary research group, which included experts with relevant expertise. One member was the PI on a prior project that translated the patient version into Norwegian (Better treatment of psychosis study—PI: Torleif Ruud). Subsequently, we obtained input from end-users, including panel groups and representatives from next-of-kin organizations, prior to conducting a pilot test with 15 relatives. Lastly, this finalized measure was employed in the IFIP study (involving patient-relative pairs), alongside the Norwegian patient version of collaboRATE and other questionnaires. Following this, we translated the slightly changed and new version of collaboRATE for relatives into English (see Table 1). The Norwegian version was used in all parts of this study.

**Table 1:** Comparison of collaboRATE for relatives in this study and the original US version.

Original/published version: collaboRATE for individuals acting on behalf of patients	New Norwegian version translated to English: collaboRATE for Next of Kin Acting to Support Patient Autonomy
1. How much effort was made to help you understand the patient's health issues?	1. Same
2. How much effort was made to listen to the things that matter most to you about the patient's health issues?	2. How much effort was made to listen to the things that *you think matter most* about the patient's health issues?
3. How much effort was made to include what matters most to you in choosing what to do next for the patient?	3. How much effort was made to include what *you know* matters most to *the patient* in choosing what to do next*?*

#### Carer well-being and support questionnaire part b (CWS)

The Carer Well-Being and Support questionnaire (CWS) short version part B—support (31)—measures the satisfaction with professional support among family carers of people with mental health problems or dementia. The CWS consists of 18 questions that respondents answer in terms of degree of satisfaction, ranging from very dissatisfied (score 0) to somewhat dissatisfied (score 1), somewhat satisfied (score 2), and very satisfied (score 3). This instrument has been shown to have acceptable reliability and validity (31).

We used four content-matching questions from CWS to assess concurrent/convergent validity with the collaboRATE items and sum scores, as depicted in Table 2.

**Table 2:** Questions in carer well-being and support questionnaire part B used to assess associations with collaboRATE for relatives.

	collaboRATE for relatives	Carer Well-Being and Support questionnaire (CWS)
A	*Information given*	
	Item 1: How much effort was made to help you understand the patient's health issues?	*How satisfied are you with the information given:* Item 1: That you have enough information about the condition/illness of the person you care for, that you feel confident in caring for them? Item 2: That you have enough information about how their condition/illness is likely to develop in the longer term?
B	*Involvement in treatment and care planning*	
	Item 2: How much effort was made to listen to the things that you think matter most about the patient's health issues? Item 3: How much effort was made to include what you know matters most to the patient in choosing what to do next?	*How satisfied are you with*: Item 9: Your involvement in important decisions (e.g., medication, hospitalization)? Item 10: Your ability to influence important decisions?
C	*Sum scores*	
	All three collaboRATE items (total range 0-27)	CWS questions 1, 2, 9 and 10 (total range 0-12)

#### Experience of caregiving (ECI)

To test discriminant validity, we correlated collaboRATE scores for relatives with scores on the content-mismatching subscales of the ECI, which is an adequately validated instrument for assessing relatives’ experience of caregiving (32, 33). On the ECI, relatives responded to 66 questions about their thoughts and judgments regarding their family member's treatment at the CMHC in the previous month, with items answered on a five-point Likert scale ranging from 1 (never) to 5 (almost always). We excluded five subscales from the ECI because they focused on similar or overlapping themes with those measured by collaboRATE for relatives (e.g., problems with healthcare services). We retained four subscales of the ECI for use in discriminant validity analysis: patient difficult behavior, which consists of eight items; patient negative symptoms (six items); patient need for backup or help (six items); and patient losses (13 items).

### Tests and statistics

To test reliability in terms of the internal consistency of collaboRATE for relatives, we calculated Cronbach's alpha at baseline and at 6-month follow-up, using 0.7 as a benchmark value of adequate consistency for the alpha coefficient (34). We used factor analysis to evaluate whether to reduce the three-item instrument to two items by examining item communalities and factor loadings. We also report mean item scores and total scores on collaboRATE for relatives at each measurement point.

Before addressing convergent and divergent validity, we used linear mixed models to assess the degree to which study site (*n* = 15) influenced variation in total scores on the collaboRATE for relatives. In this analysis, variance between the clusters (sites) was given by the intercept estimate, while the residual provided an estimate of the variance between individuals within sites. The percentage of variance in collaboRATE for relatives scores attributable to study site (the cluster intraclass correlation coefficient, ICC) was calculated as: variance between sites/(variance between sites + variance within sites).

To investigate question 2 regarding the convergence between the collaboRATE for relatives scores and comparable items and sum scores for the CWS, we used the Pearson correlation coefficient *r*. As shown in Table 2, in analysis A on “information given” we correlated collaboRATE item 1 with CWS items 1 and 2 (separately and together). For analysis B on “involvement in treatment and care planning”, we summed the collaboRATE scores for items 2 and 3 and correlated these scores with the sum scores of CWS items 9 and 10. In analysis C on “sum scores”, we correlated the sum scores of the three items on collaboRATE with the sum scores of the four selected items on CWS.

To address question 3 regarding discriminant validity, we used Pearson's *r* to test for associations between sum scores on collaboRATE for relatives and scores on each of the four ECI subscales. Because we identified small negative correlations between the constructs, we used partial correlation tests to see if controlling for third variables reduced these correlations. Specifically, we controlled for study arm and for family involvement and family psychoeducation during the 12 months prior to baseline and the 6 months prior to the 6-month follow-up.

The analyses for research questions 1 through 3 were first performed for all relatives from both study arms considered together. In subsequent sensitivity analyses, we repeated the analyses separately for relatives in each study arm.

A small set of participants with missing data on CWSs or ECIs were excluded from tests of research questions 2 and 3.

To control for Type I error, we used the Bonferroni correction for multiple testing, adjusting the significance threshold to *p* = 0.05/10 = 0.005 for research question 2 and *p* = 0.05/8 = 0.0063 for research question 3.

We studied the fourth research question only at baseline. This question looked at differences in collaboRATE scores between relatives of patients in compulsory care and relatives of patients in voluntary care. We used independent-samples *t*-tests to compare the total scores and single-item scores between the two groups. Only 19 out of 198 relatives at baseline cared for patients in compulsory care. Because of this small sample size, these analyses have low statistical power and should be viewed as exploratory.

## Results

Table 3 shows the mean item and sum scores on collaboRATE for relatives at baseline and 6-month follow-up.

**Table 3:** Scores on collaboRATE for relatives at baseline and 6-month follow-up.

CollaboRATE items and sum scores	Baseline (mean, SD) (*n* = 198)	6-month follow-up (mean, SD) (*n* = 173)
1. How much effort was made to help you understand the patient's health issues? (0-9)	5.4 (2.9)	5.5 (2.9)
2. How much effort was made to listen to the things that matter most to you about the patient's health issues? (0-9)	5.9 (2.8)	5.8 (2,8)
3. How much effort was made to include what matters most to you in choosing what to do next for the patient? (0-9)	5.8 (2.7)	5.6 (2.8)
Sum scores collaboRATE (0-27)	17.0 (7.8)	16.9 (8.2)

### Internal consistency of the new measure “collaboRATE for Next of Kin acting to support patient autonomy”

The internal consistency of the three-item collaboRATE scale for relatives was adequate in terms of Cronbach's alpha values. This was seen both for the overall sample (alpha from 0.91-0.95) and for relatives in the intervention arm (alpha from 0.89-0.96) and relatives in the control arm (alpha from 0.94-0.95), respectively (Table 4).

**Table 4:** Chronbach's alpha and mean inter-item correlations of the three-item CollaboRATE for relatives scale.

Time point	Chronbach's alpha			Mean inter-item correlation		
	All	Intervention	Control	All	Intervention	Control
Baseline	0.91	0.89	0.94	0.78	0.73	0.85
6-month follow-up	0.95	0.96	0.95	0.87	0.89	0.86

**Table 5:** ICC values and variance explained for study site (cluster) upon total scores on collaboRATE for relatives.

Time point	Between site (cluster) variance	Within site variance	ICC-value, variance explained
Baseline	0.881	59.911	0.014, 1.4%
6-month follow-up	0.001	66.725	0.0001, 0.01%

Using principal component analysis to assess item redundancy across the overall sample, we found a single factor with an eigenvalue greater than 1.0 at each measurement point. Furthermore, all three items showed high communalities (above 0.80) and strong factor loadings (above 0.90) on this factor.

### Testing effects of study site (cluster) upon scores on collaboRATE for relatives

Linear mixed models indicated that study site (cluster) had a negligible impact on the variance in total scores on collaboRATE for relatives (Table 5). The cluster ICC values were 0.014 at baseline (1.4% of variance explained by site) and <0.001 at the 6-month follow-up (essentially 0% of variance explained by site). Due to this negligible impact, we did not include study site as a covariate in the further analysis.

### Concurrent validity

Each of the three items on the new collaboRATE measure for relatives correlated positively with selected matching items on the CWS at baseline and at 6-month follow-up (cf. Table 2), with correlation coefficients ranging from *r* = 0.49 to *r* = 0.61 (Table 6A). Similar positive correlations were evident between the sum score on collaboRATE for relatives and the sum score of the CWS items at both baseline (*r* = 0.61) and 6-month follow-up (*r* = 0.66). All 10 correlations remained significant after Bonferroni corrections (*p* < 0.005).

**Table 6:** Correlation tests (Pearson *r*) for collaboRATE for relatives (CB) vs. selected items on the Carer Well-Being and Support questionnaire (CWS).

	Overall sample		Intervention arm		Control arm	
Tests	Baseline (*n* = 193)	6-month follow-up (*n* = 172)	Baseline (*n* = 117)	6-month follow-up (*n* = 105)	Baseline (*n* = 76)	6-month follow-up (*n* = 67)
*A Information given*						
CB item 1 vs. CWS item 1	*r* = 0.55*	*r* = 0.61	*r* = 0.49	*r* = 0.60	*r* = 0.49	*r* = 0.60
CB item 1 vs. CWS item 2	*r* = 0.49	*r* = 0.52	*r* = 0.45	*r* = 0.48	*r* = 0.45	*r* = 0.48
CB item 1vs CWS items 1 + 2	*r* = 0.55	*r* = 0.60	*r* = 0.49	*r* = 0.58	*r* = 0.49	*r* = 0.58
*B Involvement in treatment care and planning*						
CB items 2 + 3 vs. CWS items 9 + 10	*r* = 0.55	*r* = 0.61	*r* = 0.47	*r* = 0.65	*r* = 0.64	*r* = 0.55
*C Sum scores*						
CB sum of 3 items vs. CWS sum of 4 items	*r* = 0.61	*r* = 0.66	*r* = 0.54	*r* = 0.67	*r* = 0.71	*r* = 0.64

* = All correlations in the table are significant at *p* < 0.001.

In sensitivity analysis, the correlations between scores on collaboRATE for relatives and CWS scores in the subgroups of relatives in the intervention and control arms, respectively, were generally in line with the correlations observed in the overall sample (Table 6).

### Discriminant validity

Considering relatives from both study arms together, the sum score on collaboRATE for relatives was not positively associated with any of the four selected subscales of the ECI at baseline or at the 6-month follow-up (Table 7). Instead, all 8 correlations were slightly negative, ranging from *r* = −0.09 to *r* = −0.27, with 5 of these correlations reaching (unadjusted) statistical significance (*p* < 0.05). Three of the 8 negative correlations, all at baseline, remained significant after Bonferroni adjustment (*p* = 0.0063). This pattern of small negative correlations was not affected by controlling for possible third variables (see Methods—Tests and statistics) in partial correlation tests.

**Table 7:** Discriminant validity tests for the collaboRATE for relatives (sum scores) vs. subscales of the Experience of Caregiving Inventory (ECI)—overall sample.

CollaboRATE sum scores vs. subscales on the Experience of caregiving inventory (ECI):	Baseline (*n* = 193)	6-month follow-up (*n* = 172)
ECI Difficult behaviors	*r* = −0.25, *p* < 0.001	*r* = −0.09, *p* = 0.22, ns
ECI Negative symptoms	*r* = −0.23, *p* = 0.001	*r* = −0.10, *p* = 0.18, ns
ECI need to back-up	*r* = −0.27, *p* < 0.001	*r* = −0.18, *p* = 0.02
ECI loss	*r* = −0.17, *p* = 0.02	*r* = −0.09, *p* = 0.25, ns

In subsequent sensitivity analysis, the pattern of small, negative correlations between sum scores on collaboRATE for relatives and ECI subscale scores was observed separately in both the intervention and control samples (Tables 8, 9).

**Table 8:** Sensitivity analysis—relatives in experimental arm only. Discriminant validity tests for the collaboRATE for relatives (sum scores) vs. subscales of the Experience of Caregiving Inventory (ECI).

CollaboRATE sum scores vs. subscales on the ECI:	Baseline (*n* = 117)	6-month follow-up (*n* = 105)
ECI Difficult behaviors	*r* = −0.24, *p* = 0.008	*r* = −0.03, *p* = 0.76, ns
ECI Negative symptoms	*r* = −0.22, *p* = 0.017	*r* = −0.10, *p* = 0.31, ns
ECI need to back-up	*r* = −0.25, *p* = 0.008	*r* = −0.20, *p* = 0.04
ECI loss	*r* = −0.20, *p* = 0.035	*r* = −0.11, *p* = 0.27, ns

**Table 9:** Sensitivity analysis—relatives in the control arm only. Discriminant validity tests for the collaboRATE for relatives (sum scores) vs. subscales of the Experience of Caregiving Inventory (ECI).

CollaboRATE sum scores vs. subscales on the ECI:	Baseline (*n* = 76)	6-month follow-up (*n* = 67)
ECI Difficult behaviors	*r* = −0.26, *p* = 0.024	*r* = −0.18, *p* = 0.14, ns
ECI Negative symptoms	*r* = −0.24, *p* = 0.039	*r* = −0.11, *p* = 0.37, ns
ECI need to back-up	*r* = −0.29, *p* < 0.009	*r* = −0.14, *p* = 0.25, ns
ECI loss	*r* = −0.13, *p* = 0.26, ns	*r* = −0.06, *p* = 0.66, ns

#### Comparing collaboRATE scores for relatives to patients in compulsory vs. voluntary care

At baseline, scores on the CollaboRATE for relatives were overall lower for 19 relatives of patients in compulsory care than for 179 relatives of patients in voluntary care. The difference was significant (*p* < 0.05, unadjusted) in independent-sample t-tests for total scores on collaboRATE for relatives and for two out of three single item scores, with a similar trend observed for the third item (Table 10).

**Table 10:** CollaboRATE scores for relatives of patients in compulsory care compared with those in voluntary care.

CollaboRATE measure	Compulsory care (*n* = 19). Mean (SD)	Voluntary care (*n* = 179). Mean (SD)	*t*-test statistics
Total score	13.1 (7.7)	17.4 (7.7)	*t* = −2.3, *p* = 0.021
Item 1	3.8 (3.1)	5.5 (2.9)	*t* = −2.4, *p* = 0.019
Item 2	4.6 (3.0)	6.0 (2.8)	*t* = −2.1, *p* = 0.033
Item 3	4.7 (2.6)	5.9 (2.6)	*t* = −1.9, *p* = 0.058

## Discussion

We found that the new collaboRATE measure of SDM for relatives acting to support patient autonomy had excellent internal consistency, with Cronbach's alpha at two measurement points ranging from 0.91 to 0.95. Comparable Chronbach's alpha values for the collaboRATE measure for relatives were seen in the intervention and control arms separately. This implies that the three items of the instrument were adequately associated with the same construct. High alpha levels raise the possibility of redundant items; however, factor analysis did not suggest that any items were redundant. This aligns with the original patient version of collaboRATE, which is well-validated with three items while remaining very brief. Hence, we retained all three items in the new collaboRATE measure for relatives.

In correlating the item and sum scores on the collaboRATE for relatives with scores on matching items from the CWS, we found a moderate, positive association across all 10 tests. All 10 tests remained significant after correcting for multiple comparisons. Moreover, correlations comparable to those seen in the overall sample were observed in each of the two study arms considered separately. We expected moderate rather than high correlations between the collaboRATE for relatives and the CWS for three reasons. First, the constructs addressed by the two instruments are not fully convergent. Second, they phrase questions differently, focusing on how much was done (collaboRATE for relatives) versus how satisfied the respondent was (CWS) regarding interactions with health personnel. Third, the scope of mental health services evaluated also differs; collaboRATE for relatives focuses on the health care unit or professional that currently provides care for the patient (the CMHC in our study), whereas the CWS encompasses support more generally from any public or private health care service. These three factors would be expected to attenuate the correlations between the instruments. Therefore, the consistent finding of moderate correlations supports the validity of collaboRATE as a measure of SDM for relatives.

Discriminant validity analyses revealed a lack of positive associations between the collaboRATE for relatives scores for SDM and the relatives’ evaluations of the patients on the four selected subscales of the ECI. This was observed in the entire sample, as well as in the intervention and control arms separately. This finding supports the discriminant validity of collaboRATE for relatives. However, in the overall sample, we observed weak but significant negative correlations between the collaboRATE scale for relatives and the ECI subscales on 5 of 8 tests. While this was reduced to 3 of 8 tests after Bonferroni correction, it could indicate a small degree of inverse overlap between the measured constructs, suggesting a limitation in the discriminant analysis. Alternatively, a small negative empirical association may exist between the constructs. One possibility is that a third variable simultaneously affects relatives’ experience of SDM and their experience of the patient, as measured by the ECI. However, in partial correlation analyses, these small negative correlations remained when we controlled for study arm, family involvement, and participation in family psychoeducation. Our inability to account for the small negative correlations observed in a subset of the discriminant validity tests indicates that these tests should be interpreted with caution.

Relatives of patients in compulsory care may experience more challenges with SDM than relatives of patients in voluntary care and therefore score lower on collaboRATE for relatives. This hypothesis was supported by the 25% lower collaboRATE for relatives total scores when patients were in compulsory care compared to voluntary care—a pattern also mirrored in the relatives’ scores on the three individual collaboRATE items. These exploratory analyses support the validity of collaboRATE for relatives.

### Strengths and limitations

To the best of our knowledge, this is the first validation study of an SDM measure for relatives who support patient autonomy through supported decision-making. The collaboRATE measure for relatives was developed and pilot-tested with end users, making it a strong match for the corresponding patient measure (35). Using other validated measures at the same time allowed us to assess internal consistency, concurrent validity, and discriminant validity. Study strengths include testing across two partly different subsamples assessed at different time points from the IFIP trial, as well as running sensitivity analysis for each study arm independently.

The study, using retrospective trial data, lacked simulations or extra data to test other psychometric properties of collaboRATE for relatives, such as test-retest reliability and predictive validity. This limits our conclusions about internal consistency. Additionally, the study lacked adequate power for subgroup analyses by relationship type (e.g., spouses, parents, siblings, adult children), which limits generalizability. Another limitation is that respondents assessed SDM over the past 6 months. Because collaboRATE items are sensitive to recent experiences, the tool may work better for very recent clinical encounters. Finally, our divergent validity analysis showed small negative associations instead of the expected zero associations. This remains unexplained and points to possible issues in the divergent validity set-up.

## Conclusion

We have presented and validated the first SDM measure for relatives acting to support patient autonomy. The study provides results and methodological approaches for further validation. These tests can include other patient groups, treatment settings, languages, and compulsory mental healthcare treatment, as well as further checks for validity and reliability.

Our data on internal consistency and concurrent validity support the use of the new collaboRATE measure to assess SDM from the perspective of relatives contributing to supported decision-making for adult patients with severe and prolonged illnesses. The divergent validity of CollaboRATE for relatives was only partly confirmed, requiring further validation. An important advantage of this measure is its broad applicability regardless of whether the patient is competent to consent or has a fluctuating competency level. Furthermore, the measure can be used in contexts where relatives function as surrogate decision-makers, provided the aim remains to promote patient autonomy when the adult patient lacks capacity. Finally, it may be used alongside a validated corresponding collaboRATE measure for the patient, both in research and in routine healthcare delivery.

## Data Availability

The datasets used and analyzed in the current study are available from the IFIP trial group upon reasonable request. Requests should be directed to Reidar Pederesen, reidar.pedersen@medisin.uio.no. The collaboRATE for Next of Kin Acting to Support Patient Autonomy is subject to a Creative Commons License (CC BYNC-SA 4.0 https://creativecommons.org/licenses/by/4.0/). All enquiries about the License should be directed to the creators (RP, LH, MR, IN, KSH, Kristiane Myckland Hansson, Torleif Ruud and Glyn Elwyn).
